# The Use of DArTseq Technology to Identify Markers Related to the Heterosis Effects in Selected Traits in Maize

**DOI:** 10.3390/cimb45040173

**Published:** 2023-03-23

**Authors:** Jan Bocianowski, Agnieszka Tomkowiak, Marianna Bocianowska, Aleksandra Sobiech

**Affiliations:** 1 Department of Mathematical and Statistical Methods, Poznań University of Life Sciences, Wojska Polskiego 28, 60-637 Poznan, Poland; 2 Department of Genetics and Plant Breeding, Poznań University of Life Sciences, Dojazd 11, 60-632 Poznan, Poland; 3 Faculty of Chemical Technology, Poznań University of Technology, Piotrowo 3A, 60-965 Poznan, Poland

**Keywords:** *Zea mays* L., association analysis, molecular markers, DArTseq, heterosis

## Abstract

Spectacular scientific advances in the area of molecular biology and the development of modern biotechnological tools have had a significant impact on the development of maize heterosis breeding. One technology based on next-generation sequencing is DArTseq. The plant material used for the research consisted of 13 hybrids resulting from the crossing of inbred maize lines. A two-year field experiment was established at two Polish breeding stations: Smolice and Łagiewniki. Nine quantitative traits were observed: cob length, cob diameter, core length, core diameter, number of rows of grain, number of grains in a row, mass of grain from the cob, weight of one thousand grains, and yield. The isolated DNA was subjected to DArTseq genotyping. Association mapping was performed using a method based on the mixed linear model. A total of 81602 molecular markers (28571 SNPs and 53031 SilicoDArTs) were obtained as a result of next-generation sequencing. Out of 81602, 15409 (13850 SNPs and 1559 SilicoDArTs) were selected for association analysis. The 105 molecular markers (8 SNPs and 97 SilicoDArTs) were associated with the heterosis effect of at least one trait in at least one environment. A total of 186 effects were observed. The number of statistically significant relationships between the molecular marker and heterosis effect varied from 8 (for cob length) and 9 (for yield) to 42 (for the number of rows of grain). Of particular note were three markers (2490222, 2548691 and 7058267), which were significant in 17, 8 and 6 cases, respectively. Two of them (2490222 and 7058267) were associated with the heterosis effects of yield in three of the four environments.

## 1. Introduction

The continuous development of molecular biology and statistical tools to analyze the large amount of data obtained for different mapping populations is changing the approach to selecting plant materials for breeding [1]. There is an increasing emphasis on the selection of multiple functional traits using, for this purpose, unrelated but genetically aligned plant materials. For maize, yield and its structural traits are the most important [2,3,4]. Increasing the range of maize cultivation is linked to breeding progress, which includes exploiting heterosis (the vigor of the first generation of hybrids) and creating hybrids with lower climatic requirements [5,6,7]. Access to increasingly modern cultivation technologies and breeding methods is also very important. The demand for new varieties of maize is constantly growing, making it the subject of intensive breeding and genetic research. The identification of molecular markers linked to genes determining not only the heterosis effect or grain yield but also other functional traits is now a priority in maize breeding programs [1,8].

To identify markers and their coupled genes, association mapping [9,10] and genomic selection [11,12] are currently the most commonly used. In association mapping, we can distinguish between candidate gene association and genome-wide association study (GWAS). In candidate gene association, correlations between DNA polymorphisms in a specific gene and a trait are checked. In the absence of detailed biochemical knowledge related to the sought-after trait, a GWAS analysis is justified. This approach searches for trait–marker associations across the genome and assumes that there are markers within the genome conditioning the expression of the trait that show coupling imbalances [13]. It was on maize that the first association mapping was performed [14] using simple RAPD and RFLP techniques. With the development of new biotechnological methods and statistical programs, the number of species studied has increased.

The breakthrough came in 2006 with Illumina’s launch of the Solexa (Genome Analyzer) Next Generation Sequencer. The sequencing technology used in this device is still being developed and Illumina now has several sequencing platforms, including two for ‘bulk’ analysis (NovaSeq and NextSeq), and the smallest is for sequencing small numbers of samples with shorter genomes (MiniSeq and iSeq). The platforms differ in the amount of data that they can obtain (from 1.8 GB for MiniSeq to 6000 GB on the NovaSeq platform) and the number of reads that they can generate in a limited time [15]. Initially, the problem was the large amount of sequencing data preventing bioinformatics analyses on desktop computers; nowadays, analyses are performed on high-throughput servers with large RAM capacity, in either a Linux or an iOS environment [16]. Many bioinformatics tools run algorithms that allow whole genomes and transcriptomes to be assembled from short sequences [17]. As the results show, bioinformatics analyses currently pose the greatest difficulty in adapting NGS for diagnostics, as the result of improper folding is false positives and incorrectly folded genomes, making it difficult to correctly interpret the results obtained [18].

One technology based on next-generation sequencing is DArTseq. This technology was used in the present study to identify single nucleotide polymorphism (SNP) and Silico DArT polymorphisms, for which associations with the heterosis effect were sought. DArTseq technology (in opposition to GBS) provides a large pool of both SNP and SilicoDArT markers (which are also characterized by dominance) [19]. In this method, the complexity of the genome is reduced via restriction enzymes and sequencing short reads. DArTseq technology replaces the hybridization step, and sequencing is conducted using the Illumina system [20].

Molecular analyses in maize are focused on the identification of new markers (SNP and SilicoDArT) and quantitative trait loci (QTLs) regions, as well as breeders’ interest in DNA analyses, which additionally includes the search for new methods to select the parental components for heterosis crosses [21]. The idea here is to find a relationship between the yield of an F_1_ hybrid and the heterogeneity of loci markers for its parental forms. It is well known that breeding success is determined by access to starting materials with significant genetic diversity because well-identified and heterotically partitioned starting material results in lower costs for the entire hybrid breeding process [22].

The purpose of this study was to identify molecular markers (SNPs and SilicoDArTs) linked to the heterosis effects for yield and yield traits in maize (*Zea mays* L.).

## 2. Materials and Methods

### 2.1. Plant Material

**Figure 1:**
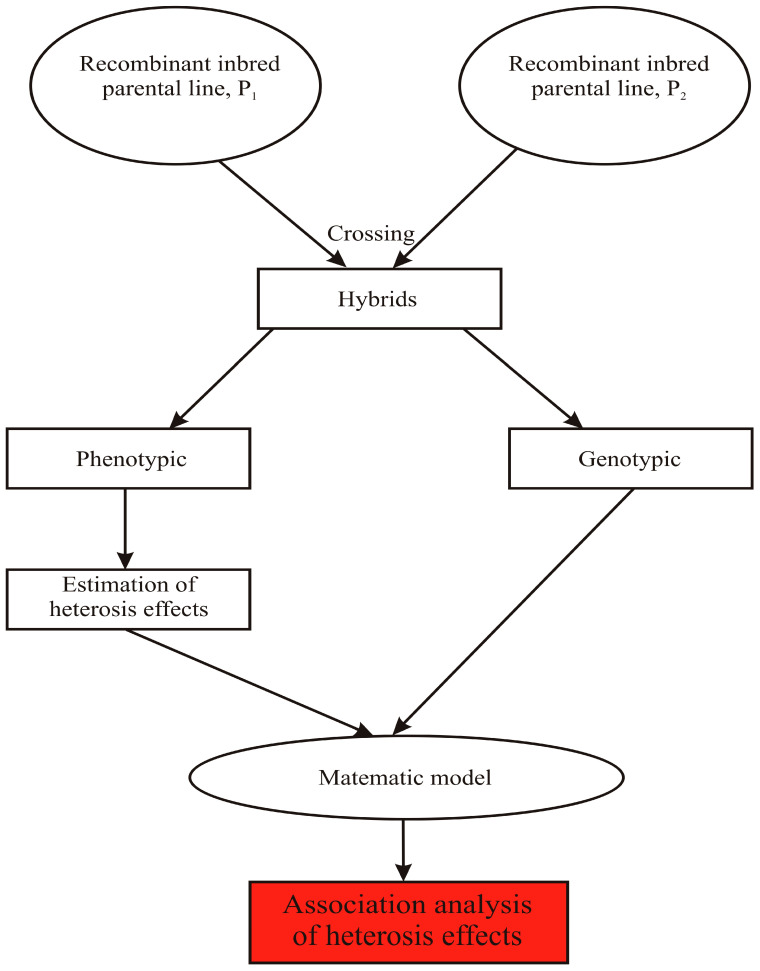
The plan of work.

The plant material for the study consisted of 13 (Bejm, Blask, Brda, Budrys, Grom, Kozak, M Glejt, M Prosna, M Wilga, Narew, O Glejt, Popis and Smok) hybrids created by crossing inbred maize lines. Maize lines and hybrids came from Smolice Plant Breading Company. The plan of the work is shown in Figure 1.

### 2.2. Field Experiment

Two-year (2013 and 2014) field experiments with hybrids were established at two breeding stations belonging to Smolice Plant Breeding, part of the Plant Breeding Acclimatization Institute Group, in Smolice (51°42′20.813″ N, 17°9′57.405″ E) and Łagiewniki (50°47′27″ N, 16°50′40″ E), Poland, in plots of 10 m^2^ in a randomized block design with three replicates. Łagiewniki and Smolice are 137 km apart. Both locations were chosen because they differ in soil type and rainfall. In Łagiewniki, the soil quality classification is predominantly class I to III, while in Smolice it is class III to V. The average annual rainfall in Łagiewniki is 560–660 mm, while in Smolice it is 390–460 mm. The locations, which vary in terms of environmental conditions, were chosen to test the influence of the environment on the magnitude of the heterosis effect. Maize was sown in each year of the study on April 20. Mineral fertilization was adapted to the needs of maize grown for grain harvest. Polyphoska in the quantity of 350 kg ha^–1^ and 160 kg ha^–1^ of urea were applied. The nutrient content was as follows: Before winter ploughing: N—21 kg N ha^–1^ in the form of polyphoska, P—70 kg P_2_O_5_ ha^−1^ in the form of polyphoska, and K—105 kg K_2_O ha^–1^ in the form of polyphoska. Before sowing: N—73.6 kg N ha^–1^ in the form of urea—was applied. The chemical control of weeds was performed with a field sprayer. Within each repetition, one cob each was selected from ten plants and biometric measurements were made on them. These biometric measurements were carried out in the first half of November each year. Nine quantitative traits were observed: cob length (LC), cob diameter (DC), core length (LCO), core diameter (DCO), the number of rows of grain (NRG), the number of grains in a row (NGR), mass of grain from the cob (MGC), weight of 1000 grains (WTGs) and yield.

### 2.3. Genotyping as Well as SNP and SilicoDArT Data Processing

The seeds of each hybrid were divided. One part was allocated to set up a field experiment and ten pieces were allocated for molecular analyses. Seeds of the F_1_ generation hybrids (10 from each hybrid) were sown onto sterile Petri plates to obtain young seedlings. Approximately 1 cm^2^ of leaf tissue was taken from each of the 10 plants and a collection sample was prepared (separately for each hybrid). The plant material prepared in this way was isolated. Isolation was carried out using Maxwell equipment from Promega, for automatic DNA isolation. The Maxwell^®^ RSC Tissue DNA Kit AS1610 was used for this purpose. After isolation, the concentration and purity of the DNA samples were tested and equal dilutions were prepared to 100 ng µL^–1^. DNA concentration and purity were checked using a DeNovix spectrophotometer from TK Biotech. Care was taken to only select samples for sequencing where the DNA purity at 260/280 absorbance was between 1.8 and 2.0 and at 230/260 absorbance was not less than 1.8. DNA prepared in this way was placed in a 96-well plate and sent for sequencing to Diversity Arrays Technology.

For next-generation sequencing, F_1_ hybrids and inbred lines (highly homozygous), which were the parental forms of these hybrids, were sent to a 96-well plate. However, for the above publication, only the sequencing results of the F_1_ hybrids were used from the database of the results obtained. The results for the lines were used for a different inference. The inbred lines were obviously characterized by low yield because they had previously undergone inbreeding to obtain plant materials that were highly homozygous. The F_1_ hybrids were characterized by a high yield.

The genotypic data used for association mapping were obtained from polymorphisms identified in DArT and candidate gene sequences. The details of DArT sequences were described by Tomkowiak et al. [23]. Genotyping using DArTseq technology was performed by Diversity Arrays Technology Pty Ltd. (Kilian A., Canberra, Australia). DNA sample digestion/ligation reactions were processed according to Kilian and Graner [20], but by replacing a single PstI-compatible adaptor with two adaptors corresponding to PstIand NspI-compatible sequences and moving the assay on the sequencing platform as described by Sansaloni et al. [24]. The PstI-compatible adapter was designed to include an Illumina flow cell attachment sequence, a sequencing primer sequence and a “staggered” varying length barcode region, similar to the sequence reported by Elshire et al. [25]. The reverse adapter contained a flow cell attachment region and an NspI-compatible overhang sequence. Only “mixed fragments” (PstI–NspI) were amplified via PCR using the following reaction conditions: denaturation for 1 min at 94 °C; followed by 30 cycles of 94 °C for 20 s, 57 °C for 30 s and 72 °C for 45 s; and the final elongation at 72 °C for 7 min. After PCR, equimolar amounts of amplification products from each sample of the 96–well microtiter plate were bulked and applied in c–Bot (Illumina) bridge PCR, followed by sequencing on Illumina Hiseq2500. The sequencing (single read) was run for 78 cycles. Sequences generated from each lane were processed using proprietary DArT analytical pipelines. In the primary pipeline, the fastq files were first processed to filter away poor-quality sequences, applying more stringent selection criteria to the barcode region compared to the rest of the sequence. In this way, the assignments of the sequences to specific samples carried in the “barcode split” step were very reliable. Approximately 2,500,000 (±7%) sequences per barcode/sample were used in the marker calling. Finally, identical sequences were collapsed into “fastqcall files”. These files were used in the secondary pipeline for DArT PL’s proprietary SNP and SilicoDArT (presence/absence of restriction fragments in representation) calling algorithms (DArTsoft14).

### 2.4. Statistical Analysis

**Figure 2:**
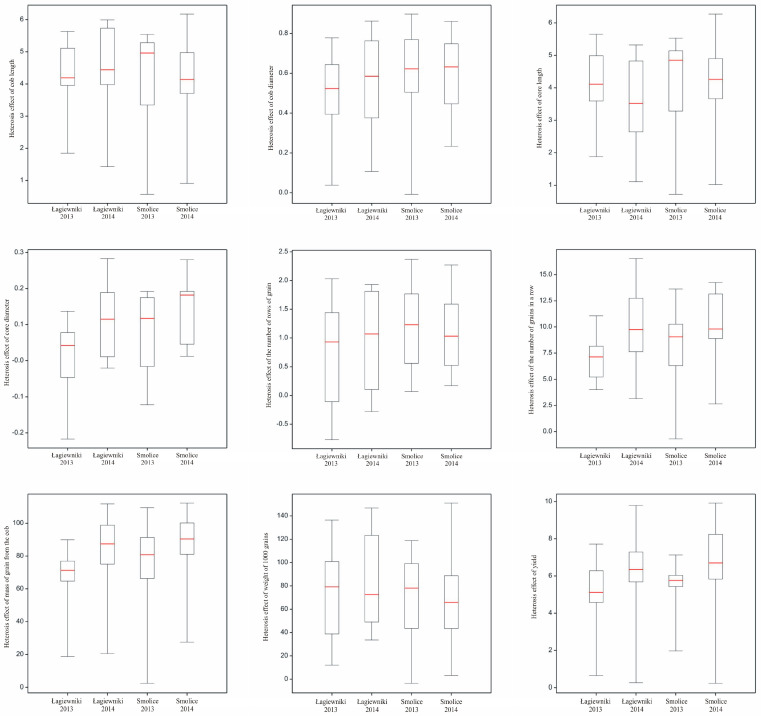
Box-and-whisker diagrams of the values of heterosis effects of nine quantitative traits; classified by the studied environments.

Heterosis effects for hybrids were calculated relative to the average of both parents, for each trait (Figure 2) [23].

The effects of heterosis were tested at the 0.05 level. DArT sequences meeting three criteria were selected for association analysis: the missing observation fractions < 10%, minor allele frequency (MAF) > 0.25 and one SNP and SilicoDArT within a given sequence (69 nt). Association mapping of heterosis effects was performed using a method based on a mixed linear model [26,27]. The GenStat v. 22 [28] statistical software package was used for all of the analyses. The significance of associations between SilicoDArT and SNP markers and heterosis effects was tested at the 0.001 level (with Benjamini–Hochberg correction, for multiple comparisons) [29]. All analyses were conducted for four environments (two localities, two years) independently.

## 3. Results

**Figure 3:**
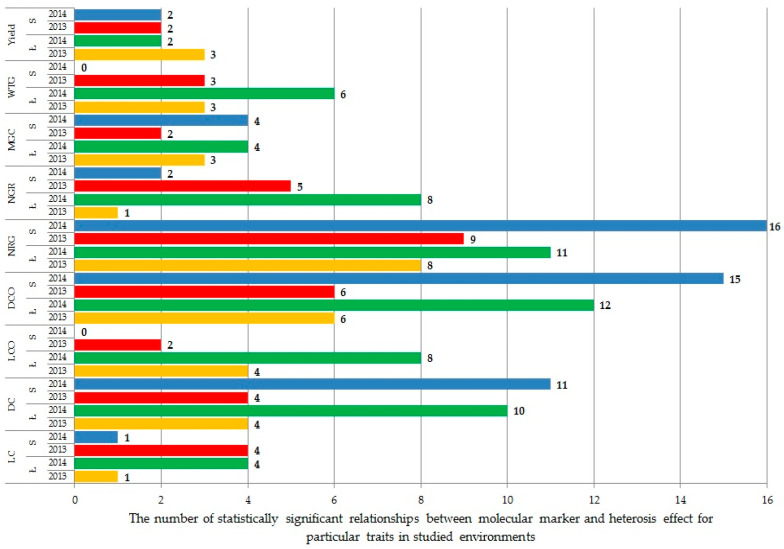
The number of statistically significant relationships between the molecular marker and heterosis effect for particular traits in studied environments. LC—cob length, DC—cob diameter, LCO—core length, DCO—core diameter, NRG—the number of rows of grain, NGR—the number of grains in a row, MGC—mass of grain from the cob, WTGs—weight of 1000 grains, Ł—Łagiewniki, S—Smolice.

Next-generation sequencing yielded 81,602 molecular markers (28,571 SNPs and 53,031 SilicoDArTs). Of the 81,602, 15,409 (13,850 SNPs and 1559 SilicoDArTs) were selected for association analysis using the criteria given above. Due to statistically significant interactions, association analyses of genotypes–years, genotypes–locations, years–locations and genotypes–years–locations were performed independently for four environments (combinations of years–locations). The 105 molecular markers (8 SNPs and 97 SilicoDArTs) were associated with the heterosis effect of at least one trait in at least one environment. A total of 186 effects were observed (Figure 3, Table 1, Table 2, Table 3, Table 4, Table 5, Table 6, Table 7, Table 8 and Table 9).

The heterosis effect of the cob length was determined by five markers: four SilicoDArTs and one SNP (Table 1). The effects of these markers varied from 2.494 (for 9693403—SilicoDArT marker in Łagiewniki 2014) to 3.579 (for SilicoDArT marker 2490222 in Smolice 2013), with an average of 2.977. The percentage variance accounted by individual markers varied from 59.4% (for 4584773—SilicoDArT marker in Smolice 2014) to 72.3% (for SilicoDArT marker 2548691 in Łagiewniki 2014), with an average of 66.76%. SilicoDArT marker 2490222 was associated with the heterosis effect of cob length in all four environments (Table 1). The other four markers determined the heterosis effect only in single environments. The largest number (four) of associations was observed in Łagiewniki in 2014.

**Table 1:** Molecular markers significantly (LOD > 3.0) associated with the heterosis effect of cob length (LC).

Type of Marker	Marker Name	Łagiewniki				Smolice			
		2013		2014		2013		2014	
		Effect	R^2^	Effect	R^2^	Effect	R^2^	Effect	R^2^
SilicoDArT	2490222	2.626	70.4	3.394	69.2	3.579	70.2	3.5	72
SilicoDArT	2548691			2.964	72.3				
SilicoDArT	9693403			2.494	60.1				
SilicoDArT	4584773							2.754	59.4
SNP	4776193			2.502	60.5				

R^2^—percentage variance accounted [in %].

The heterosis effect of cob diameter was determined by 18 SilicoDArT markers (Table 2). The effects of these markers varied from −0.3455 (for marker 4580744 in Smolice 2014) to 0.606 (for marker 2490222 in Smolice 2013), with a mean value of 0.367. R^2^ for individual markers ranged from 58.3% (for marker 21697645 in Smolice 2013) to 74.5% (for marker 4769006 in Łagiewniki 2014), with an average of 63.91%. SilicoDArT markers 21697645 and 2529741 were associated with the heterosis effect of cob length in three of the four environments (except for Łagiewniki 2014 and Smolice 2013, respectively). The largest number (11) of associations was observed in Łagiewniki in 2014 (Table 2).

**Table 2:** Molecular markers (SilicoDArT) significantly (LOD > 3.0) associated with the heterosis effect of cob diameter (DC).

Marker Name	Łagiewniki				Smolice			
	2013		2014		2013		2014	
	Effect	R^2^	Effect	R^2^	Effect	R^2^	Effect	R^2^
9626263			0.4121	61.1			0.3547	72.1
2455130			0.4016	60.9				
2380663			0.4261	69.7			0.3279	63.8
7057126			0.4267	66.2			0.3295	61
4769006			0.4387	74.5			0.3401	69.3
21697645	0.3414	63			0.432	58.3	0.3582	65.5
4584035			0.4293	59.5				
4766435			0.4349	69.1				
2529741	0.3228	62.6	0.4079	59.7			0.3248	59
7051437							0.3237	62
100000032							0.3255	62.8
100000044			0.3945	58.4				
2490222	0.4276	60.1			0.606	71.8		
4583359					0.444	62		
9698148			0.419	67.1			0.326	63
4584773	0.3664	60.2			0.513	70		
9679877							0.3455	60.3
4580744							−0.3455	60.3

R^2^—percentage variance accounted [in %].

The heterosis effect of core length was determined by eleven markers: eight SilicoDArTs and three SNPs (Table 3). The effects of these markers ranged from −2.241 (for 4767009—SilicoDArT marker in Łagiewniki 2014) to 3.377 (for 2490222—SilicoDArT marker in Smolice 2013), with an average of 2.033. The R^2^ for particular markers ranged from 58.7% (for SilicoDArT marker 2383880 in Łagiewniki 2013 and SNP marker 4592834 in Łagiewniki 2014) to 78.1% (for SilicoDArT marker 7058267 in Smolice 2013), with an average of 63.57%. No marker was significant for the heterosis effect in Smolice in 2014. No marker was significant for the heterosis effect in more than two environments. Three markers (SilicoDArT markers 2490222 and 2548691, and SNP marker 4776193) were associated with the heterosis effect of core length in two environments (Table 3). The largest number (eight) of associations was observed in Łagiewniki in 2014.

**Table 3:** Molecular markers significantly (LOD > 3.0) associated with the heterosis effect of core length (LCO).

Type of Marker	Marker Name	Łagiewniki				Smolice			
		2013		2014		2013		2014	
		Effect	R^2^	Effect	R^2^	Effect	R^2^	Effect	R^2^
SilicoDArT	9624336			2.138	59.5				
SilicoDArT	2490222	2.446	62.7			3.377	65.3		
SilicoDArT	2548691	2.108	63.6	2.608	63.8				
SilicoDArT	2383880	1.763	58.7						
SilicoDArT	2425331			2.241	62.6				
SilicoDArT	4767009			−2.241	62.6				
SilicoDArT	4764251			2.207	64				
SilicoDArT	7058267					3.13	78.1		
SNP	4592834			2.126	58.7				
SNP	4776193	1.895	61.5	2.409	65.5				
SNP	7049788			2.254	63.4				

R^2^—percentage variance accounted [in %].

The heterosis effect of core diameter was determined by 27 markers: 24 SilicoDArTs and 3 SNPs (Table 4). The effects of individual markers ranged from −1.1633 (for 2552284—SilicoDArT marker in Łagiewniki 2014) to 0.2323 (for 2495461—SilicoDArT marker in Smolice 2013), with an average of 0.1091. The R^2^ for particular markers ranged from 58.3% (for 21698399—SilicoDArT marker in Smolice 2014) to 83.3% (for 4769006—SilicoDArT marker in Smolice 2014), with an average of 65.98%. The SNP marker 4590442 was associated with the heterosis effect of core diameter in all four environments (Table 4). Two SilicoDArT markers (4769006 and 21697628) were associated in three of the four environments. The largest number (15) of associations was observed in Smolice in 2014.

**Table 4:** Molecular markers associated with the heterosis effect of core diameter (DCO).

Type of Marker	Marker Name	Łagiewniki				Smolice			
		2013		2014		2013		2014	
		Effect	R^2^	Effect	R^2^	Effect	R^2^	Effect	R^2^
SilicoDArT	4590044			0.1531	58.9				
SilicoDArT	9626263							0.1409	61.6
SilicoDArT	2380663							0.1411	65.2
SilicoDArT	4588725			0.1651	70			0.1423	66.5
SilicoDArT	9680027					0.1816	62.1		
SilicoDArT	4769006	0.1595	65.3	0.1649	69.8			0.1573	83.3
SilicoDArT	21698399							0.1377	58.3
SilicoDArT	2602895							0.1426	66.9
SilicoDArT	21697628	0.1668	60.6	0.1762	68.2	0.2039	67.8		
SilicoDArT	4766435			0.1605	62.1				
SilicoDArT	2529741	0.1603	62.4						
SilicoDArT	4583359	0.1744	67.1			0.2202	80.5		
SilicoDArT	9698148			0.1574	62.8				
SilicoDArT	4590586							0.1346	58.5
SilicoDArT	2495461	0.1905	66.7			0.2323	74		
SilicoDArT	4778477			0.1655	66.6				
SilicoDArT	9679877							0.1503	63.2
SilicoDArT	100000188					0.1858	65.3	0.1372	61.2
SilicoDArT	9713962			0.1556	61.2				
SilicoDArT	4580744							−0.1503	63.2
SilicoDArT	2504629							−0.1467	59.8
SilicoDArT	2464213			−0.1531	58.9				
SilicoDArT	2552284			−0.1633	68.3			−0.1369	60.9
SilicoDArT	4591821			−0.1589	60.7				
SNP	7058274							0.1467	59.8
SNP	4777654							−0.1426	66.9
SNP	4590442	0.1642	65.9	0.1712	72	0.2075	79.3	0.1595	81.4

R^2^—percentage variance accounted [in %].

The 34 SilicoDArT markers and one SNP marker were associated with the heterosis effect of the number of rows of grain (Table 5). The effects of the individual markers ranged from –1.412 (for 4765009—SilicoDArT marker in Łagiewniki 2014) to 1.597 (for 2544571—SilicoDArT marker in Łagiewniki 2014), with an average of 0.583. The R^2^ for individual markers ranged from 58.2% (for 4764693—SilicoDArT marker in Smolice 2013 and 2393601 SilicoDArT marker in Łagiewniki 2014) to 79.9% (for 2544571 SilicoDArT marker in Łagiewniki 2014), with an average of 65.42%. No marker was significant for the heterosis effect in all four environments. Only one marker (SilicoDArT 2437173) was associated with the heterosis effect of the number of rows of grain in three environments (except for Łagiewniki 2013). The largest number (16) of associations was observed in Smolice in 2014 (Table 5).

**Table 5:** Molecular markers associated (LOD > 3.0) with the heterosis effect of the number of rows of grain (NRG).

Type of Marker	Marker Name	Łagiewniki				Smolice			
		2013		2014		2013		2014	
		Effect	R^2^	Effect	R^2^	Effect	R^2^	Effect	R^2^
SilicoDArT	7057126							1.025	59.5
SilicoDArT	2379644							1.01	60.9
SilicoDArT	4778753	1.309	61.5						
SilicoDArT	2544571			1.597	79.9				
SilicoDArT	4764693	1.316	58.9			1.168	58.2		
SilicoDArT	2491739	1.491	69.4			1.326	69		
SilicoDArT	2437173			1.412	68.2	1.233	65.9	1.055	63.5
SilicoDArT	4581103					1.156	60.2		
SilicoDArT	100000044			1.32	61.8			1.054	67
SilicoDArT	100000049					1.201	62		
SilicoDArT	2444826							1.09	68.4
SilicoDArT	2435684							1.201	61.5
SilicoDArT	9668509	1.322	62.9	1.404	71.1				
SilicoDArT	9698148							1.02	62.2
SilicoDArT	4588300			1.379	68.3			1.072	69.7
SilicoDArT	4765474			−1.379	68.3			−1.072	69.7
SilicoDArT	2462176	1.334	64.2						
SilicoDArT	9706913							1.08	67.1
SilicoDArT	2386741					1.15	59.4		
SilicoDArT	9624718					1.181	63.1		
SilicoDArT	5584758	1.479	76.7						
SilicoDArT	2443558			1.325	58.9				
SilicoDArT	2611917							1.16	70
SilicoDArT	2393601			1.286	58.2				
SilicoDArT	21696515			1.499	78.1				
SilicoDArT	7048312	1.384	69.8						
SilicoDArT	4591958							−1.201	61.5
SilicoDArT	4765009			−1.412	68.2				
SilicoDArT	2425024							−1.08	67.1
SilicoDArT	2386050							−1.16	70
SilicoDArT	4578555			−1.32	61.8			−1.054	67
SilicoDArT	4583930					−1.187	63.9		
SilicoDArT	4578963							−1.01	60.9
SilicoDArT	4591690	−1.297	60.2						
SNP	4764456					−1.221	64.4		

R^2^—percentage variance accounted [in %].

The heterosis effect of the number of grains in a row was determined by 15 SilicoDArT markers (Table 6). The effects of individual markers ranged from −6.98 (for 2532754 marker in Smolice 2013) to 8.55 (for 2490222 marker in Smolice 2013), with an average of 5.725. The R^2^ for individual markers ranged from 58.1% (for 4769448 marker in Łagiewniki 2014) to 76.7% (for 7058267 marker in Smolice 2013), with an average of 63.43%. Only one marker (2490222) was associated with the heterosis effect of the number of grains in a row in two of the four environments (Smolice in both years). The other 14 markers determined the heterosis effect of the number of grains in a row only in single environments. The largest number (eight) of associations was observed in Łagiewniki in 2014 (Table 6).

**Table 6:** Molecular markers (SilicoDArT) significantly (LOD > 3.0) associated with the heterosis effect of the number of grains in a row (NGR).

Marker Name	Łagiewniki				Smolice			
	2013		2014		2013		2014	
	Effect	R^2^	Effect	R^2^	Effect	R^2^	Effect	R^2^
2456387			6.16	61.2				
2434942			6.35	58.3				
4765710					6.98	67.9		
4778447			5.89	58.5				
2400466			6.59	63.3				
2490222					8.55	61.6	7.99	65.8
2548691			7.32	65.5				
4578219	3.422	59.5						
4575644					6.25	59.4		
2547687			6.18	65.2				
2523729			6.15	64.5				
2562473							6.65	61.5
4769448			6.02	58.1				
2532754					−6.98	67.9		
7058267					8.07	76.7		

R^2^—percentage variance accounted [in %].

The heterosis effect of mass of grain from the cob was determined by seven SilicoDArT markers (Table 7). The effects of individual markers ranged from 35.98 (for 2548691 marker in Łagiewniki 2013) to 72.60 (for 2548691 marker in Smolice 2013), with an average of 52.682. The R^2^ for individual markers ranged from 59.0% (for 2434942 marker in Łagiewniki 2014) to 86.7% (for 2490222 marker in Smolice 2014), with an average of 68.78%. SilicoDArT marker 2490222 was associated with the heterosis effect of mass of grain from the cob in all four environments, whereas marker 2548691 was associated in three of the four environments, except for Smolice 2013 (Table 7). The largest number (four) of associations was observed in Łagiewniki in 2014 and in Smolice in 2014.

**Table 7:** Molecular markers (SilicoDArT) significantly (LOD > 3.0) associated with the heterosis effect of mass of grain from the cob (MGC).

Marker Name	Łagiewniki				Smolice			
	2013		2014		2013		2014	
	Effect	R^2^	Effect	R^2^	Effect	R^2^	Effect	R^2^
2434942			46.5	59				
2397282			51.5	60.4			48	59.2
2490222	47.95	82.4	67.75	79.3	72.6	78.8	66.41	86.7
2548691	35.98	61.1	54	67.4			50	65
4584773							50.5	66.3
4778649	36.37	62.7						
7058267					57.3	65.8		

R^2^—percentage variance accounted [in %].

The heterosis effect of weight of one thousand grains was determined by ten SilicoDArT markers (Table 8). The effects of individual markers ranged from –69.6 (for 4594009 marker in Łagiewniki 2013) to 69.7 (for 100000034 marker in Łagiewniki 2013), with an average of 20.2. The R^2^ for individual markers ranged from 58.3% (for 4779015 marker in Łagiewniki 2014) to 74.0% (for 4594009 marker in Łagiewniki 2013), with an average of 62.66%. No marker was significant for the heterosis effect of WTGs in Smolice in 2014. No marker was significant for the heterosis effect of WTGs in more than two environments. Two markers (100000034 and 4594009) were associated with the heterosis effect of WTGs in two environments (Table 8). The largest number (six) of associations was observed in Łagiewniki in 2014.

**Table 8:** Molecular markers (SilicoDArT) significantly (LOD > 3.0) associated with the heterosis effect of weight of 1000 grains (WTGs).

Marker Name	Łagiewniki				Smolice			
	2013		2014		2013		2014	
	Effect	R^2^	Effect	R^2^	Effect	R^2^	Effect	R^2^
4773439			61.4	59.4				
100000034	69.7	60.3			69	60.8		
4779015			61	58.3				
100000073			−65.6	61.2				
2429497					58	59.9		
100000117			62.5	65.3				
2526480			61.2	58.8				
4594009	−69.6	74			−66.7	69.3		
2449821			−61.2	58.8				
4587345	62.7	65.8						

R^2^—percentage variance accounted [in %].

The heterosis effect of yield was determined by five markers: four SilicoDArTs and one SNP (Table 9). The effects of individual markers ranged from 2.729 (for 9693146 SilicoDArT marker in Smolice 2013) to 6.850 (for 2490222 SilicoDArT marker in Smolice 2014), with an average of 4.830. The R^2^ for individual markers ranged from 58.7% (for 2548691 SilicoDArT marker in Łagiewniki 2014) to 78.1% (for 2490222 SilicoDArT marker in Łagiewniki 2014), with an average of 68.7%. SilicoDArT markers 2490222 and 7058267 were associated with the heterosis effect of yield in three of the four environments, except for Smolice 2013 (Table 9). The other three markers determined the heterosis effect of yield only in single environments. The largest number (three) of associations was observed in Łagiewniki in 2013.

**Table 9:** Molecular markers associated with the heterosis effect of yield.

Type of Marker	Marker Name	Łagiewniki				Smolice			
		2013		2014		2013		2014	
		Effect	R^2^	Effect	R^2^	Effect	R^2^	Effect	R^2^
SilicoDArT	9693146					2.729	61.7		
SilicoDArT	2490222	4.896	73.3	6.509	78.1			6.85	76.8
SilicoDArT	2548691	3.804	58.7						
SilicoDArT	7058267	3.809	58.9	5.18	66.1			5.766	73.9
SNP	7054002					3.926	70.8		

R^2^—percentage variance accounted [in %].

Of particular note are three markers (2490222, 2548691 and 7058267) which were significant in 17, 8 and 6 cases, respectively. Two of them (2490222 and 7058267) were associated with the heterosis effects of yield in three of the four environments (Table 9).

## 4. Discussion

It is estimated that by 2050 the planet will be inhabited by approximately 10 billion people and therefore there is increasing pressure to increase and balance food production. Breeders, with the support of scientific entities, are developing ever newer tools that guarantee ever greater accuracy in selection [30,31]. Current selection methods have been enriched by statistical models and advances in molecular biology that allow for both the identification of markers for individual traits that are the result of single genes and those that are determined by multiple QTLs to different degrees, explaining the phenotypic variation in a trait [32,33]. Recent selection methods allow work to be carried out using both Mendelian and population-based models. Each of the aforementioned models has a defined field of action that defines its use within specific experimental needs. Increasingly, molecular markers obtained by next-generation sequencing and association mapping are being used for selection [34].

Association mapping is one tool for identifying novel markers and associated genes. Association mapping assumes that multiple plant materials (usually unrelated or distantly related with a high degree of alignment within lineages) are taken for study [35]. Obviously, the size of the study population depends on the species and trait. In order to achieve the desired effect, the size of the mapping population can vary from about 200 to 800 individuals [35]. Increasing the population size is usually not economically justified [36]. The use of diverse plant materials means that recombinations occurring at earlier stages of derivation of materials are frequent, and the variation contained between them reflects the variation in the gene pool [35]. Thus, the trait markers identified should be useful for broader plant material. The method assumes that all analyzed plant materials are screened for a specific trait and are profiled with the relevant DNA markers [37]. In order to identify trait markers, it is necessary to have a large number of markers densely and evenly distributed across the genome. The density of such coverage is dependent on the value of the coupling imbalance, which will depend on the species and the trait [38]. As a result, markers obtained by next-generation sequencing methods such as GBS [25] or DArTseq [39] and relatively high computing power [38] are required for such studies. In this study, association mapping was used to identify molecular markers related with the heterosis effect. The results of our study indicate that the 105 molecular markers (8 SNPs and 97 SilicoDArTs) were associated with the heterosis effect of at least one trait in at least one environment. A total of 186 effects were observed. The number of statistically significant relationships between the molecular marker and heterosis effect varied from eight (for cob length) and nine (for yield) to forty-two (for the number of rows of grain).

Heterosis, as a genetic phenomenon, determines the beneficial consequence of crossbreeding, which is the vigor of the first generation of hybrids [40]. Interest in hybrid vigor and its practical use started in 1880, when Beal found and described the existence of hybrid vigor after crossing two population varieties. Very often, the economic importance of the heterosis phenomenon can be demonstrated without statistical analyses, as its symptoms are sometimes striking [41]. Although the phenomenon of heterosis has measurable economic benefits, unfortunately it cannot be perpetuated. One exception is vegetatively propagated plants, e.g., bananas, where production has been dominated by two vegetatively propagated hybrids [42].

Very often, but not always, the phenomenon of heterosis concerns quantitative traits, e.g., plant yield. A major breeding goal is the heterosis of grain yield, although we know little about how heterosis effects affect yield components [43]. Many authors argue that grain yield is a trait characterized by low heritability and that the lack of correlation between yield levels of parental lines and their F1 generation hybrids is a result of gene dominance and overdominance [44,45]. In some experimental combinations, despite the significance of additive effects, the percentage of non-additive gene effects to the genetic yield variance of F_1_ generation hybrids often exceeded 80%. It appeared that environmental interaction effects influenced the expression of gene action [46]. In the past decade, many researchers in their studies [47,48,49] have applied molecular biology methods to detect and locate loci determining grain yield and yield structure traits in maize. However, intensive research is still underway to search for loci that are coupled to yield and its components [50]. According to various studies, QTL regions associated with genes that determine grain yield and its components are distributed throughout the genome [4]. Our results indicate that five molecular markers (four SilicoDArTs and one SNP) were associated with the heterosis effect of yield. Two (SilicoDArTs: 2490222 and 7058267) of them were associated with the heterosis effect of yield in three of the four environments.

In maize research, molecular analyses intensively focus on identifying QTL regions and new markers. Breeders with DNA analyses are also interested in developing methods to select parental components for heterosis crosses [21] and to predict the effect of heterosis [51]. An idea here is to find an association between the heterogeneity of loci markers for their parental forms and the yield of an F_1_ hybrid [52]. It is well known that starting materials with high genetic diversity make for breeding success, as well-identified and heterotically divided starting material results in lower costs for the entire hybrid breeding process [22]. We can divide plant materials into heterotic groups according to the following criteria: genetic origin (pedigree), results of crossing in diallel systems, geographical origin and molecular markers. Unfortunately, the first three subdivision criteria presented are flawed. Therefore, in recent years, attempts have been made to select parental components for heterosis crosses on the basis of their genetic diversity, determined by molecular markers [53]. In the research presented here, association analysis was used to detect relationships between the effect of heterosis and the genotype of hybrids. Similar studies were conducted on winter oilseed rape [54].

Rapidly developing new genotyping methods based on hybridization markers or next generation sequencing are finding increasing application in basic research. The reproducibility of the results of DArT and DArTseq technologies and the availability of a very large number of SNP markers, as well as their declining costs, mean that these modern methods are finding increasing applications in identifying markers for quantitative traits and genome-wide selection in economically important plants. The use of these methods reduces work time significantly. DArTseq technology also proves itself as a powerful diagnostic tool for studying genotypic diversity [55]. DArT and DArTseq technology have been successfully applied in Chinese common wheat (*Triticulum aestivum* L.) to study the population structure and genetic diversity for 111 breeding lines and cultivars from northern China. The results obtained provided valuable information into China’s wheat breeding program for selecting parental forms [56]. The DArT method has been widely used in parentage analysis, such as in oats (*Avena* sp.), where groups corresponding to spring and winter forms were distinguished for 134 varieties [57]. A low degree of differentiation was shown in 232 forms of Bengal pea (*Cajanus cajan*) based on 696 DArT markers, of which only 64 were polymorphic; wild forms were the most diverse [58]. Tomkowiak et al. [53], using next-generation sequencing and association mapping, identified 15,409 silicoDArT markers and SNPs significantly associated with yield and yield structure traits in maize. From these markers, 18 SilicoDArT markers determined the quantitative trait, in all localities considered. A physical map was constructed based on these markers. Six of the eighteen identified markers (numbered 1818, 14506, 2317, 3233, 11657 and 12812) were located within the genes on chromosomes 8, 9, 7, 3, 5 and 1, respectively. Two markers (no. 15097—SilicoDArT—and no. 5871—SNP) linked to fusarium resistance in maize have been localized within genes [59] on chromosomes 2 and 3, respectively. In their study, Tomkowiak et al. [60], using DArTseq technology and association mapping, identified six markers associated with vigor and germination in maize. The existing literature indicates that four of these genes (phosphoinositide phosphatase sac7 isoform ×1 gene, grx_c8-glutaredoxin subgroup iii gene, sucrose synthase 4 isoform ×2 gene and putative SET domain-containing protein family isoform ×1 gene) determine the level of seed germination and seed vigor in maize.

## 5. Conclusions

The integration of molecular genetics with traditional plant selection methods guarantees an objective assessment of phenotype, taking into account environmental factors, and offers the possibility of eliminating undesirable genotypes as early as the seedling stage, thus reducing breeding time. The development of molecular biology, including in vitro culture breeding methods, marker technologies (e.g., next-generation sequencing), the construction of genetic maps of crop plants of different species or the use of genetic mapping, quantitative trait mapping and, more recently, association mapping in combination with appropriately selected mapping populations and automated plant phenotyping methods, open up new possibilities for breeding. The association mapping used in the present study is a good and useful tool for identifying molecular markers that determine the effect of heterosis yield and yield structure traits in maize. The main objective of our work was to propose a method for detecting markers coupled to the effects of heterosis of quantitative traits. The results obtained show the versatility of the given markers as tools for heterosis diagnosis. The advantage of the proposed approach is that it is independent of the sign of the heterosis effect.

## Data Availability

The data presented in this study are available on request from the corresponding author.
